# The Surgery of the Skull and Brain

**Published:** 1913-03

**Authors:** 


**The Surgery of the Skull and Brain.** By L. Bathe Rawling, F.R.C.S. Pp. xi, 340. London: Oxford Medical Publications. 1912. Price, 25s. net.

This work is one by an author who has devoted himself for many years to the study of a special subject, and to its practical exposition. It is marked by a lucidity of description and illustration which makes it easy to read, and which carry conviction to the reader.

The chapter on fractures of the skull is particularly valuable, and the original illustrations in this and other parts of the book make it a most welcome addition to the literature of the subject.

The author adopts and recommends a simple operative technic, preferring hand instruments to those actuated by mechanical motors.

We should welcome in future editions a chapter on the physiology of the brain in its relations to practical surgical diagnosis and treatment.

